# The IDA Peptide Controls Abscission in *Arabidopsis* and *Citrus*

**DOI:** 10.3389/fpls.2015.01003

**Published:** 2015-11-19

**Authors:** Leandro H. Estornell, Mari Wildhagen, Miguel A. Pérez-Amador, Manuel Talón, Francisco R. Tadeo, Melinka A. Butenko

**Affiliations:** ^1^Centre de Genómica, Institut Valencià d’Investigacions AgràriesMontcada, Spain; ^2^Section for Genetics and Evolutionary Biology, Department of Biosciences, University of OsloOslo, Norway; ^3^Instituto de Biología Molecular y Celular de Plantas, Universidad Politécnica de Valencia–Consejo Superior de Investigaciones CientíficasValència, Spain

**Keywords:** IDA, HAESA, receptor-like kinases, *Citrus*, floral abscission, fruit production, crop yield

## Abstract

Organ abscission is an important process in plant development and reproduction. During abscission, changes in cellular adhesion of specialized abscission zone cells ensure the detachment of infected organs or those no longer serving a function to the plant. In addition, abscission also plays an important role in the release of ripe fruits. Different plant species display distinct patterns and timing of organ shedding, most likely adapted during evolution to their diverse life styles. However, it appears that key regulators of cell separation may have conserved function in different plant species. Here, we investigate the functional conservation of the citrus ortholog of the *Arabidopsis* peptide ligand INFLORESCENCE DEFICIENT IN ABSCISSION (AtIDA), controlling floral organ abscission. We discuss the possible implications of modifying the citrus IDA ortholog for citrus fruit production.

## Introduction

Small peptides are used as important signaling ligands to control plant growth and development and more than 1000 genes encoding putative peptides have been discovered in *Arabidopsis thaliana* (*Arabidopsis*; Lease and Walker, 2006; Murphy et al., 2012; Grienenberger and Fletcher, 2015). One such peptide ligand, INFLORESCENCE DEFICIENT IN ABSCISSION (AtIDA), was found to be important for the regulation of floral organ abscission in *Arabidopsis* as the *ida* mutant failed to abscise its floral organs (Butenko et al., 2003). In wild type (wt) plants the abscission process takes place at the boundary between the organ to be shed and the main plant body, in cell files constituting the abscission zone (AZ). After the formation of an AZ, which occurs early and simultaneously with the development of lateral organs from the apical meristem, the AZ cells can be distinguished from their neighbors by being small, densely cytoplasmic and lacking vacuoles (Addicott, 1982; Sexton and Roberts, 1982; Osborne, 1989; Roberts et al., 2000; Liljegren, 2012). Once the abscission process is initiated these cells go through three sequential developmental stages; (i) cell fate determination and acquisition of competence to respond to abscission signals; (ii) cell wall loosening and expansion by cell wall remodeling (CWR) enzymes followed by organ separation; and (iii) differentiation of a protective lignified layer (Patterson, 2001; Aalen et al., 2013; Gubert et al., 2014).

In the *ida* mutant, the cell separation event fails to take place (Butenko et al., 2003). In accordance with this, an IDA overexpression line (35S:AtIDA) exhibited ectopic abscission and displayed an enlarged floral AZ in *Arabidopsis* (Stenvik et al., 2006). Additionally, organ loss was observed at the bases of the pedicel, branches of the inflorescence, and cauline leaves; places where abscission normally does not occur in *Arabidopsis*. AtIDA signaling and the 35S:AtIDA phenotypes are dependent on the two leucine-rich repeat (LRR) receptor-like kinases (RLKs) HAESA (HAE) and HAESA-LIKE 2 (HSL2; Jinn et al., 2000; Cho et al., 2008; Stenvik et al., 2008). Upon receptor activation a MAP kinase signaling event is turned on ultimately leading to the induction of genes encoding CWR enzymes (Cai and Lashbrook, 2008; Cho et al., 2008; Kumpf et al., 2013). Recently, it was shown that a hydroxyprolinated AtIDA peptide of 12 amino acids was sufficient to mediate AtIDA signaling. Furthermore, this same peptide was shown to bind the HSL2 receptor with high affinity (Butenko et al., 2014).

The gene encoding the AtIDA peptide belongs to a family of five *AtIDA-LIKE* (*AtIDL*) genes in *Arabidopsis* that are expressed in a variety of tissues in the *Arabidopsis* plant body, including the base of the pedicel, in the floral tissue AZ, in the funicle AZ and in the main root tip (Stenvik et al., 2008). Interestingly, some of the regions of expression represent cell files where abscission takes place in other plants such as in *Citrus* (citrus) species (Lewis et al., 2006; Estornell et al., 2013), where, unlike for *Arabidopsis*, leaf abscission does take place. Flower and fruit abscission in citrus occurs at the pedicel AZ (called AZ-A), located close to the boundary between the pedicel and the twig, and at the ovary/fruit AZ (called AZ-C), located in the calyx between the pericarp and the nectary or floral disk, respectively (Tadeo et al., 2008). Cultivated citrus trees usually display heavy flowering. However, a high number of flowers and young fruits are shed during the fruit set period (physiological drop) thus maintaining only those fruits that can be nurtured until maturity. The attachment force by which a fruit is held to the calyx shows a large reduction in value at the end of the maturation phase of fruit development in almost all citrus species. However, early and mid-season varieties of sweet orange are especially prone to a premature decline in the attachment force of the fruit leading to pre-harvest fruit abscission. This problem has a serious economic impact especially in those citrus producing areas dedicated to the fresh fruit market as the Mediterranean Basin. The premature decline in the attachment force of the fruit prevents on-tree storage of fruit which shortens the harvesting season and hinders the orderly fruit marketing. Therefore, understanding the mechanisms controlling fruit abscission in citrus, with the prospect of genetic engineering (transgenic or CRISPR gene editing technologies), is of importance for two reasons; one for controlling fruit loss during the physiological drop and pre-harvest abscission, and two to facilitate shedding of unmarketable fruits remaining on the tree once the harvesting season is over.

INFLORESCENCE DEFICIENT IN ABSCISSION and the IDL peptides are evolutionary conserved across the plant kingdom and they exhibit sequence similarity in their conserved C-terminal domain (PIP domain) containing the highly active AtIDA peptide (Butenko et al., 2003; Stenvik et al., 2008; Butenko et al., 2014). Furthermore, the HSL receptors are found across the plant kingdom and in dicots the HSL receptor sequences are conserved (Stø et al., 2015). It has previously been proposed that the presence of *IDL* and *HSL* transcripts at sites where cell separation occur in *Arabidopsis* may indicate that these genes play a more general role in cell separation (Butenko et al., 2009). Here, we explore on the possibility that this may not only be the case in *Arabidopsis* but also in citrus.

We show that the C- terminal domain of AtIDA is highly conserved in the IDA and IDL citrus orthologs. We also provide evidence that the citrus IDA (CitIDA) most similar to AtIDA, CitIDA3, has a function in abscission. We discuss the potential agriculture implications of modifying CitIDA3 and additional downstream signaling components of the IDA pathway identified in *Arabidopsis*.

## Materials and Methods

### Plant Material and Generation of Transgenic Lines

The *CitIDA3* CDS was amplified from clementine cDNA, obtained from total RNA isolated from post-anthesis floral buds, by amplification of a 270 bp fragment with primers: 5′ CACCATGGCTTCTTCTTCTTCTTC 3′, 5′ TCAATTTTGAGTAGAATCCACAACAGA 3′. In parallel, screening of a clementine BAC collection (Terol et al., 2008) was carried out, and as a result BAC clones CCL005I18 and CCH3006E10 were identified to contain the genomic region of *CitIDA3*. A 3 kb *CitIDA3* promoter fragment was amplified from BAC clone CCL005I18 using 5′ CACCGAATTTGTAATTAACTTGTCTTCTT 3′ and 5′ ATAAATTGTTTGTTTTGGGTTGGC 3′ and cloned into pKGWFS7.0 (Plant Systems Biology, Ghent, Belgium). *35S:CitIDA3* lines were made by cloning the CDS fragments into pK2GW7.0 (Plant Systems Biology, Ghent, Belgium). Sixteen independent lines were obtained and two independent lines were investigated in detail. The *Agrobacterium tumefaciens* C58 strain was used for *Arabidopsis* transformation by flower dipping (Clough and Bent, 1998).

### Plant Material for Complementation of the *ida* Mutation

Two independent *35S:CitIDA3* lines were crossed into the *ida-2* mutant background (Cho et al., 2008). The F_2_ progeny of both lines were phenotypically scored based on their ability to abscise and genotyped by the *ida-2* genotyping primers 5′ CGGTGTTGGTGGATCCAAGTC 3′ and 5′ CCCTCATTTCCGCCACACTTA 3′ and the T-DNA LBb1 primer 5′ ATTTTGCCGATTTCGGAAC 3′. The presence of the *35S:CitIDA3* transgene was verified with primers: 5′ CGCACAATCCCACTATCCTT 3′, 5′ TCAATTTTGAGTAGAATCCACAACAGA 3′.

### Petal Breakstrength Measurements

The measurements were performed using a petal breakstrength meter, as previously described (Stenvik et al., 2008). The petal breakstrength was quantified as the force, in gram equivalents, required to remove a petal from a flower (Butenko et al., 2003).

### Identification of IDL and HSL Citrus Orthologs

We employed two different strategies to identify IDL citrus orthologs. We first performed TBLASTN searches against the citrus (sweet orange and clementine) genome at the Join Genome Institute (JGI^1^) using the amino acid sequences corresponding to the variable region and the EPIP motif of the *Arabidopsis* IDL proteins. Second, we used only the amino acid sequences of the EPIP motifs of the *Arabidopsis* IDL proteins as query to perform TBLASTN searches in the NCBI GenBank dbEST database for the expressed sequence tag (EST) set of citrus and *Poncirus trifoliata*. Selected EST sequences (query coverage > 85%; max identity > 55%) were downloaded from the GenBank database and assembled using the CAP3 program (Huang and Madan, 1999). We used the sequences of the unigenes to query against the citrus (sweet orange and clementine) genome at the JGI.^1^ Signal peptide predictions of all putative citrus IDL proteins were carried out using the SignalP 4.0 server^2^.

To identify the citrus HSL orthologs TBLASTN (six-frame translation) searches against the citrus (sweet orange and clementine) genome were performed at the JGI^1^ using the amino acid sequences of the *Arabidopsis* HAE (AT4G28490), HSL1 (AT1G28440), and HSL2 (AT5G65710) proteins.

### Protein Alignments and Phylogenetic Analysis

Multiple sequence alignments of the IDL and HSL proteins were performed using ClustalW tools offered by GenomeNet^3^ with default parameters and displayed with GENEDOC. Based on the aligned sequences of the citrus IDL and HSL proteins, Neighbor Joining trees were constructed using MEGA version 6.0 (Tamura et al., 2013) with a bootstrap of 1000 replicates.

## Results

### Identification of the Citrus Members of the IDA-HAE/HSL2 Signaling Module

**FIGURE 1:**
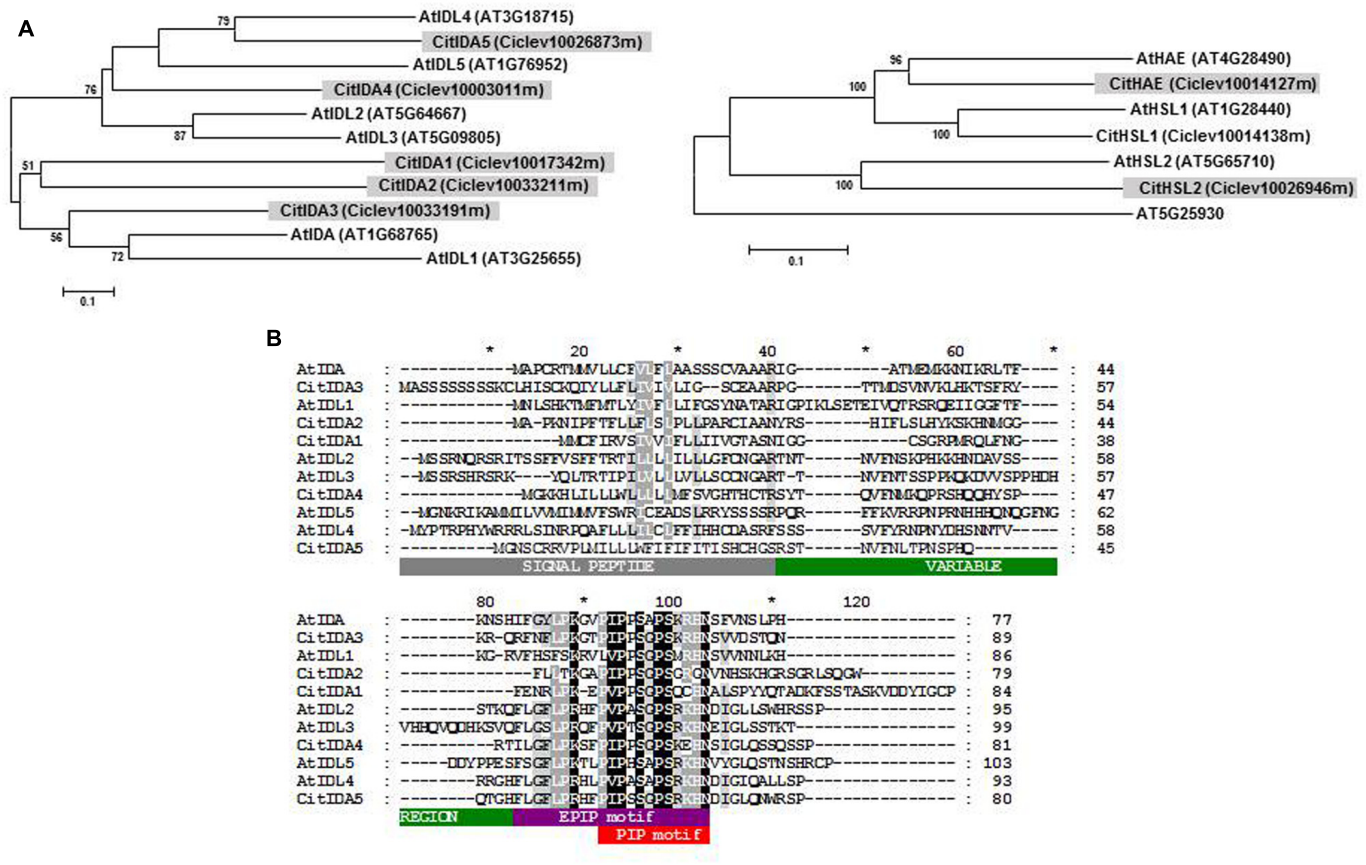
**Proteins encoded by *IDL* and *HSL* genes from *Arabidopsis* and citrus. (A)** Phylogenetic relationships between IDL and HSL proteins. At5g25930 is a protein kinase not related to HSL proteins. **(B)** Alignment of the IDL full-length proteins showing the N-terminal secretion signal, the variable region, and the short proline-rich EPIP and PIP motives.

In the citrus (sweet orange and clementine) genome five *IDL* genes were identified. They were named *CitIDA1* to *CitIDA5* depending on the location of their sequences in the citrus chromosomes or scaffolds (Supplementary Table S1). The phylogenetic relationship between the six *Arabidopsis* and five citrus IDL proteins were investigated, showing CitIDA3 to be the closest related to AtIDA (**Figure 1A**). This gave rise to the possibility that CitIDA3 could have a function in regulating cell separation during organ abscission. Supporting this idea the complete nucleotide sequence of *CitIDA3* can be reconstructed from two ESTs derived from AZ libraries (Supplementary Table S3).

All of the citrus *IDL* genes showed uninterrupted open reading frames encoding translation products with a predicted N-terminal signal peptide sequence followed by a variable region and a highly conserved C-terminal PIP domain (Stenvik et al., 2008; Butenko et al., 2014; **Figure 1B**). Within the twelve amino acid residues that make up the PIP domain, there were invariant amino acid residues (positions 3, 5, 7, 8, and 12) and others in which the substitutions were conservative (positions 1, 2, and 6). CitIDA3 was the IDA-like protein with highest similarity to the PIP motif of AtIDA differing only in one amino acid (**Figure 1B**). Given these similarities we hypothesized that CitIDA3 has a conserved function similar to that of AtIDA.

Our genome-wide analysis identified three HSL proteins in the citrus (sweet orange and clementine) genome highly similar to each of the *Arabidopsis* proteins (**Figure 1A** and Supplementary Table S2). Therefore, the IDA-HAE/HSL2 signaling module could also work in citrus.

### Function and Expression of *CitIDA3* Phenocopies *AtIDA*

**FIGURE 2:**
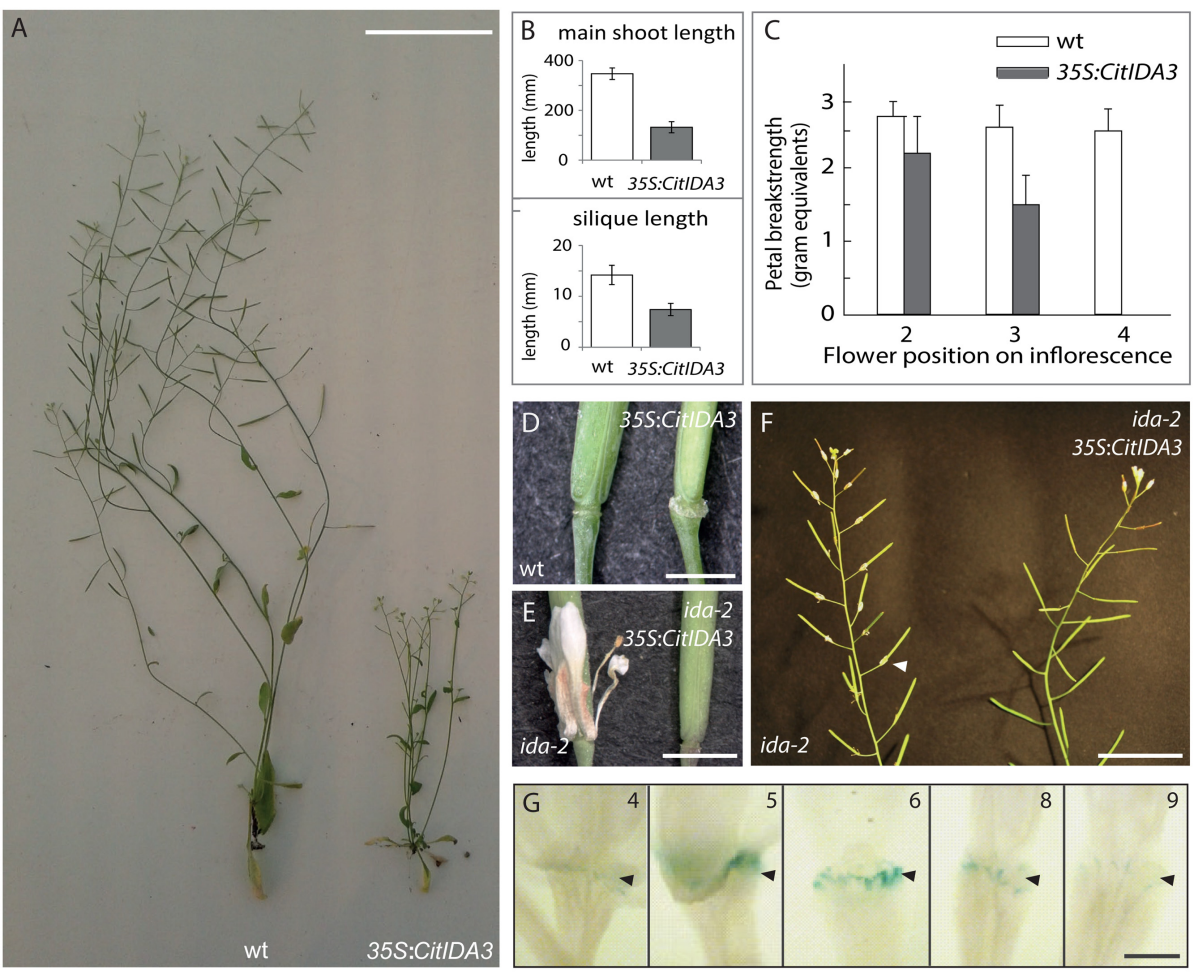
**CitIDA3 phenocopies the function of AtIDA. (A)** Phenotype of fully developed *Arabidopsis* wt (Col-0) plants and *Arabidopsis* plants ectopically overexpressing the citrus IDA ortholog, *35S:CitIDA3*. Scalebar = 5 cm. **(B)** Comparison between main shoot (upper panel) and silique (lower panel) length in wt and *35S:CitIDA3* plants. Values represent mean ± SD, *n* = 10 (main shoot length) *n* = 25 (silique length). **(C)** Petal breakstrength measurements of wt and *35S:CitIDA3* petals. Values represent mean ± SD, *n* = 15. **(D)** Comparison of wt and *35S:CitIDA3* AZ. Scalebar = 2 mm. **(E)** Comparison of *ida-2* and *ida-2 35S:CitIDA3* floral abscission. Scalebar = 3 mm. **(F)** Comparison of the inflorescence of *ida-2* and *ida-2 35S:CitIDA3.* Arrow head indicates attached petal. Scalebar = 3 cm. **(G)** *Pro_CitIDA3_:GUS* expression in *Arabidopsis* floral AZ. The numbers indicate flower position along the inflorescence, where position 1 refers to the flower at anthesis. Black arrow heads indicate the AZ. Scalebar = 1 mm.

To investigate whether *CitIDA3* has a conserved function to *AtIDA* in regulating cell separation during organ abscission, *Arabidopsis* plants were transformed with a construct driving *CitIDA3* expression with the strong constitutive cauliflower mosaic virus 35S promoter. Fully developed *35S:CitIDA3* plants showed reduced stature and shorter siliques in comparison to wild-type plants (**Figures 2A,B**). The *35S:CitIDA3* transgenic plants abscised their floral organs at an earlier stage than wild-type plants. Petal breakstrength (pBS), the force needed to remove petals from a flower, decreases along the length of the inflorescence. Changes in progression of abscission are determined by pBS (Bleecker and Patterson, 1997). In wt pBS reaches zero at floral position 8, while for *35S:CitIDA3* this occurs at position 4, comparable to that observed for *35S:AtIDA* (**Figure 2C**; Stenvik et al., 2006). Furthermore, the premature floral organ abscission observed for *35S:CitIDA* was associated with an increase in the size of the AZ similar to that observed for *35S:AtIDA* (**Figure 2D**; Stenvik et al., 2006; Shi et al., 2011).

In an additional experiment performed to investigate the spatial and temporal activity of the *CitIDA3* promoter, we monitored the expression of a *Pro_CitIDA3_:GUS* construct in *Arabidopsis* plants. In the flowers, *CitIDA3* was expressed in the style and in floral organ AZs (Supplementary Figure S1A). GUS expression was absent in flowers from positions 1–4 but detected in the AZ of flowers from positions 5–8 (**Figure 2G**). *CitIDA3* was also expressed in cotyledons, developing leaves and roots of 1-week-old transgenic *Arabidopsis* seedlings (Supplementary Figure S1B). The expression of *CitIDA3* in *Arabidopsis* flowers is similar to that of *AtIDA* (Butenko et al., 2003).

### *35SCitIDA3* Complements the Abscission Deficiency of the *ida* Mutant

In order to investigate to what extent CitIDA3 could replace the function of AtIDA, *35S:CitIDA3* plants were crossed into the *ida-2* mutant background. The F_2_ progeny of two independent lines was genotyped in order to identify *ida-2* plants. 6 out of 29 and 5 out of 27 plants were homozygous for the *ida-2* allele. Furthermore, the plants were scored for their abscission phenotype. Two and three of the *ida-2* plants from each cross, respectively, showed the *ida-2* phenotype, with attached petals after anthesis (**Figures 2E,F**), The remaining *ida-2* homozygotes showed wt abscission (**Figures 2E,F**). All of these plants contained the *35S:CitIDA3* transgene indicating that the presence of *35S:CitIDA3* is sufficient to induce abscission in *ida-2* mutant plants.

## Discussion

Pre-harvest abscission is a problem in citrus producing areas dedicated to the fresh fruit market, preventing on-tree storage of fruit and thereby shortening the harvesting and marketing season. On the other hand, the force required to remove mature fruits from the trees in citrus varieties dedicated to the processing market (juices and concentrates) is too large for effective mechanical harvesting. Therefore, new citrus genotypes with different abscission behavior or plants derived from genetic engineering (transgenic or CRISPR gene editing technologies) aiding in timing or execution of citrus fruit specific abscission would be favorable.

In *Arabidopsis*, the small peptide IDA forms a ligand-receptor module with the two RLKs HAE and HSL2 to initiate a signaling pathway in floral organ abscission (Stenvik et al., 2008). Even though IDA was first found to regulate floral organ abscission, it has later been shown to play a role in cell separation during lateral root emergence, providing this peptide with a more general function in the control of cell separation (Kumpf et al., 2013). The discovery that IDA, HSL and additional downstream components of the IDA signaling system are found across the plant kingdom (Butenko and Simon, 2015; Stø et al., 2015) makes it plausible that orthologs in citrus have a conserved function. Indeed, several auxin response factor (ARF) genes conserved between citrus and *Arabidopsis* have been shown to have a role in fruit abscission in citrus and floral abscission in *Arabidopsis* (Ellis et al., 2005; Tadeo et al., 2015). Thus, different cell separation processes appear to share common signaling elements that are conserved across plant species.

The results shown here indicate that *CitIDA3* plays a role in floral abscission when overexpressed in *Arabidopsis*. The *Arabidopsis 35S:CitIDA3* transgenic plants were characterized by reduced plant and silique growth and precocious floral organ abscission together with an increased size of the AZ (**Figures 2B–D**). Overexpression of *CitIDA3* resulted in a highly similar phenotype to that observed in *Arabidopsis 35S:AtIDA* plants (Stenvik et al., 2006). Furthermore expression of the *CitIDA3* transgene in an *ida-2* mutant background was sufficient to rescue the abscission defect of the mutant and the *Pro_CitIDA3_:GUS* expression pattern in *Arabidopsis* plants is also consistent with a role of this gene in organ abscission (**Figures 2D–G**). Additionally, we identified two LRR-RLKs highly homologous in sequence to the *Arabidopsis* HAE and HSL2 in the citrus (sweet orange and clementine) genome (**Figure 1A** and Supplementary Table S2). All these results strongly suggest that the IDA-HAE/HSL2 abscission-signaling pathway characterized in *Arabidopsis* is conserved in citrus and therefore it could be feasible to manipulate *CitIDA3* in order to prevent or stimulate organ abscission in this fruit crop.

Downstream components of the IDA-HAE/HSL2 abscission-signaling pathway could also be potential candidates for manipulation in order to control abscission in citrus. There is genetic evidence for several KNOTTED LIKE HOMEOBOX (KNOX) transcription factors working downstream of the activated IDA-HAE/HSL2 pathway. These include *BREVIPEDICELLUS (BP)/KNOTTED-LIKE FROM ARABIDOPSIS THALIANA1* (*KNAT1*), *KNAT2*, and *KNAT6* (Shi et al., 2011). There are two genes in the citrus (sweet orange and clementine) genome (LOC102615394/Ciclev10001508m and LOC102628261/Ciclev10001779m, respectively) highly homologous to *KNAT1* and *KNAT2/KNAT6* suggesting that the known downstream components of the IDA-HAE/HSL2 abscission-signaling pathway also exist in citrus.

The MADS-domain transcription factor AGAMOUS-like 15 (AGL15) that previously was reported to play a role in regulating floral abscission in *Arabidopsis* has recently been shown to act downstream of the IDA-HAE/HSL2 and MAP kinase signaling module to regulate *HAE* expression (Fernandez et al., 2000; Patharkar and Walker, 2015). Phosphorylation of AGL15 relieves repression of *HAE* expression leading to production of *HAE* transcript in a positive feedback loop, thereby increasing the expression of the receptor (Patharkar and Walker, 2015). Manipulation of a citrus version of AGL15 (LOC102612882/Ciclev10032519m) could be a target to control abscission as could the CWR enzymes induced by IDA. Several members of different classes of CWR enzymes have been found in *Arabidopsis*, tomato and citrus (Estornell et al., 2013). By investigating the enzymes that are expressed in the citrus AZs, fruit abscission prevention or stimulation could be further modified.

Considering all the above, we can glimpse the possibilities available to manipulate the expression of *CitIDA3* and/or the citrus orthologs of the downstream components of the IDA-HAE/HSL2 abscission-signaling pathway as an agronomic tool that would affect positively on the economic benefit of growers and the citrus industry in general.

## Author Contributions

LE, MW, MP-A, MT, FT, and MB designed the research. LE, MW, MP-A, MT, FT, and MB performed the research. MW, FT, and MB analyzed the data. MW, FT, and MB wrote the article.

## Conflict of Interest Statement

The authors declare that the research was conducted in the absence of any commercial or financial relationships that could be construed as a potential conflict of interest.

## Footnotes

1 http://phytozome.jgi.doe.gov/

2 http://www.cbs.dtu.dk/services/SignalP/

3 http://www.genome.jp/tools/clustalw/

## References

[B1] Aalen R. B. Wildhagen M. Sto I. M. Butenko M. A. (2013). Ida: a peptide ligand regulating cell separation processes in *Arabidopsis*. *J. Exp. Bot.* 64 5253–5261. 10.1093/jxb/ert338 24151306

[B2] Addicott F. T. (1982). *Abscission*. Berkeley, CA: University of California Press.

[B3] Bleecker A. B. Patterson S. E. (1997). Last exit: senescence, abscission, and meristem arrest in *Arabidopsis*. *Plant Cell* 9 1169–1179. 10.1105/tpc.9.7.1169 9254934 PMC156989

[B4] Butenko M. A. Patterson S. E. Grini P. E. Stenvik G.-E. Amundsen S. S. Mandal A. (2003). INFLORESCENCE DEFICIENT IN ABSCISSION controls floral organ abscission in *Arabidopsis* and identifies a novel family of putative ligands in plants. *Plant Cell* 15 2296–2307. 10.1105/tpc.014365 12972671 PMC197296

[B5] Butenko M. A. Simon R. (2015). Beyond the meristems: similarities in the CLV3 and IDA peptide mediated signalling pathways. *J. Exp. Bot.* 66 5195–5203. 10.1093/jxb/erv310 26105996

[B6] Butenko M. A. Vie A. K. Brembu T. Aalen R. B. Bones A. M. (2009). Plant peptides in signalling: looking for new partners. *Trends Plant Sci.* 14 255–263. 10.1016/j.tplants.2009.02.002 19362511

[B7] Butenko M. A. Wildhagen M. Albert M. Jehle A. Kalbacher H. Aalen R. B. (2014). Tools and strategies to match peptide-ligand receptor pairs. *Plant Cell* 26 1838–1847. 10.1105/tpc.113.120071 24808051 PMC4079353

[B8] Cai S. Lashbrook C. C. (2008). Stamen abscission zone transcriptome profiling reveals new candidates for abscission control: enhanced retention of floral organs in transgenic plants overexpressing *Arabidopsis* ZINC FINGER PROTEIN2. *Plant Physiol.* 146 1305–1321. 10.1104/pp.107.110908 18192438 PMC2259061

[B9] Cho S. K. Larue C. T. Chevalier D. Wang H. Jinn T. L. Zhang S. (2008). Regulation of floral organ abscission in *Arabidopsis thaliana*. *Proc. Natl. Acad. Sci. U.S.A.* 105 15629–15634. 10.1073/pnas.0805539105 18809915 PMC2563077

[B10] Clough S. J. Bent A. F. (1998). Floral dip: a simplified method for *Agrobacterium*-mediated transformation of *Arabidopsis thaliana*. *Plant J.* 16 735–743. 10.1046/j.1365-313x.1998.00343.x 10069079

[B11] Ellis C. M. Nagpal P. Young J. C. Hagen G. Guilfoyle T. J. Reed J. W. (2005). AUXIN RESPONSE FACTOR1 and AUXIN RESPONSE FACTOR2 regulate senescence and floral organ abscission in *Arabidopsis thaliana*. *Development* 132 4563–4574. 10.1242/dev.02012 16176952

[B12] Estornell L. H. Agusti J. Merelo P. Talon M. Tadeo F. R. (2013). Elucidating mechanisms underlying organ abscission. *Plant Sci.* 19 48–60. 10.1016/j.plantsci.2012.10.008 23265318

[B13] Fernandez D. E. Heck G. R. Perry S. E. Patterson S. E. Bleecker A. B. Fang S. C. (2000). The embryo MADS domain factor AGL15 acts postembryonically. Inhibition of perianth senescence and abscission via constitutive expression. *Plant Cell* 12 183–198. 10.1105/tpc.12.2.183 10662856 PMC139757

[B14] Grienenberger E. Fletcher J. C. (2015). Polypeptide signaling molecules in plant development. *Curr. Opin. Plant Biol.* 23 8–14. 10.1016/j.pbi.2014.09.013 25449721

[B15] Gubert C. M. Christy M. E. Ward D. L. Groner W. D. Liljegren S. J. (2014). ASYMMETRIC LEAVES 1 regulates abscission zone placement in *Arabidopsis* flowers. *BMC Plant Biol.* 14:195 10.1186/s12870-014-0195-5 PMC4223632 25038814

[B16] Huang X. Madan A. (1999). Cap3: a DNA sequence assembly program. *Genome Res.* 9 868–877. 10.1101/gr.9.9.868 10508846 PMC310812

[B17] Jinn T. L. Stone J. M. Walker J. C. (2000). Haesa, an *Arabidopsis* leucine-rich repeat receptor kinase, controls floral organ abscission. *Genes Dev.* 14 108–117. 10640280 PMC316334

[B18] Kumpf R. P. Shi C. L. Larrieu A. Sto I. M. Butenko M. A. Peret B. (2013). Floral organ abscission peptide IDA and its Hae/Hsl2 receptors control cell separation during lateral root emergence. *Proc. Natl. Acad. Sci. U.S.A.* 110 5235–5240. 10.1073/pnas.1210835110 23479623 PMC3612645

[B19] Lease K. A. Walker J. C. (2006). The *Arabidopsis* unannotated secreted peptide database, a resource for plant peptidomics. *Plant Physiol.* 142 831–838. 10.1104/pp.106.086041 16998087 PMC1630735

[B20] Lewis M. W. Leslie M. E. Liljegren S. J. (2006). Plant separation: 50 ways to leave your mother. *Curr. Opin. Plant Biol.* 9 59–65. 10.1016/j.pbi.2005.11.009 16337172

[B21] Liljegren S. J. (2012). Organ abscission: exit strategies require signals and moving traffic. *Curr. Opin. Plant Biol.* 15 670–676. 10.1016/j.pbi.2012.09.012 23047135

[B22] Murphy E. Smith S. De Smet I. (2012). Small signaling peptides in *Arabidopsis* development: how cells communicate over a short distance. *Plant Cell* 24 3198–3217. 10.1105/tpc.112.099010 22932676 PMC3462626

[B23] Osborne D. J. (1989). Abscission. *Crit. Rev. Plant Sci.* 8 103–129. 10.1080/07352688909382272

[B24] Patharkar O. R. Walker J. C. (2015). Floral organ abscission is regulated by a positive feedback loop. *Proc. Natl. Acad. Sci. U.S.A.* 112 2906–2911. 10.1073/pnas.1423595112 25730871 PMC4352813

[B25] Patterson S. E. (2001). Cutting loose. Abscission and dehisence in *Arabidopsis*. *Plant Physiol.* 126 494–500. 10.1104/pp.126.2.494 11402180 PMC1540116

[B26] Roberts J. A. Whitelaw C. A. Gonzalez-Carranza Z. H. Mcmanus M. T. (2000). Cell separation processes in plants– models, mechanisms and manipulation. *Ann. Bot. (Lond.)* 86 223–235. 10.1006/anbo.2000.1203

[B27] Sexton R. Roberts J. A. (1982). Cell biology of abscission. *Ann. Rev. Plant Physiol.* 33 133–162. 10.1146/annurev.pp.33.060182.001025

[B28] Shi C. L. Stenvik G. E. Vie A. K. Bones A. M. Pautot V. Proveniers M. (2011). *Arabidopsis* class I KNOTTED-like homeobox proteins act downstream in the Ida-Hae/Hsl2 floral abscission signaling pathway. *Plant Cell* 23 2553–2567. 10.1105/tpc.111.084608 21742991 PMC3226213

[B29] Stenvik G. E. Butenko M. A. Urbanowicz B. R. Rose J. K. Aalen R. B. (2006). Overexpression of INFLORESCENCE DEFICIENT IN ABSCISSION activates cell separation in vestigial abscission zones in *Arabidopsis*. *Plant Cell* 18 1467–1476. 10.1105/tpc.106.042036 16679455 PMC1475485

[B30] Stenvik G. E. Tandstad N. M. Guo Y. Shi C. L. Kristiansen W. Holmgren A. (2008). The EPIP peptide of INFLORESCENCE DEFICIENT IN ABSCISSION is sufficient to induce abscission in *Arabidopsis* through the receptor-like kinases HAESA and HAESA-LIKE2. *Plant Cell* 20 1805–1817. 10.1105/tpc.108.059139 18660431 PMC2518227

[B31] Stø I. M. Orr R. J. S. Fooyontphanich K. Jin X. Knutsen J. M. B. Fischer U. (2015). Conservation of the abscission signaling peptide IDA during Angiosperm evolution: withstanding genome duplications and gain and loss of the receptors HAE/HSL2. *Front. Plant Sci.* 6:931 10.3389/fpls.2015.00931 PMC4627355 26579174

[B32] Tadeo F. R. Agustí J. Merelo P. Estornell L. H. Cercós M. Terol J. (2015). “To fall or not to fall, that’s the question!” Molecular mechanisms underlying organ abscission in Citrus. *Acta Hortic.* 1065 1189–1195.

[B33] Tadeo F. R. Cercós M. Colmenero-Flores J. M. Iglesias D. J. Naranjo M. A. Ríos G. (2008). Molecular physiology of development and quality of citrus. *Adv. Bot. Res.* 47 148–202.

[B34] Tamura K. Stecher G. Peterson D. Filipski A. Kumar S. (2013). Mega6: molecular evolutionary genetics analysis version 6.0. *Mol. Biol. Evol.* 30 2725–2729. 10.1093/molbev/mst197 24132122 PMC3840312

[B35] Terol J. Naranjo M. A. Ollitrault P. Talon M. (2008). Development of genomic resources for *Citrus clementina*: characterization of three deep-coverage BAC libraries and analysis of 46,000 BAC end sequences. *BMC Genomics* 9:423 10.1186/1471-2164-9-423 PMC2561056 18801166

