# Supplementary material for: The IDA Peptide Controls Abscission in Arabidopsis and Citrus
Source: Front Plant Sci. 2015 Nov 19;6:1003. doi: 10.3389/fpls.2015.01003 (PMC4652038; doi:10.3389/fpls.2015.01003)
Supplement: Supplementary file 1 [file Data_Sheet_1.PDF]

Supplementary Table 1. Citrus transcripts, CDS sequences and deduced amino acid sequences of the members of the citrus *IDL* gene family (source: *Citrus clementina* genomic sequence at Phytozome).

Gene name<br>(Transcript name)	CDS sequence	Peptide sequence
CitIDA1<br>(Ciclev10017342m)<br>(Scaffold 2)	ATGATGTGTTTTATTAGGGTTTCTATT<br>GTCGTTATTTTCTTGTTAATTATTGTG<br>GGTACGGCTTCGAATATTGGTGGATGC<br>AGCGGCCGGCCAATGAGGCAGCTGTTC<br>AATGGTTTTGAAAACAGGCTACCAAAA<br>GAACCAGTGCCGCCTTCAGGGCCCTCT<br>CAGTGCCACAACGCCCTTAGTCCTTAC<br>TATCAAACGGCCGATAAGTTCTCTTCC<br>ACTGCCAGCAAAGTTGATGATTATATT<br>GGTTGCCCTTGA	MMCFIRVSIVVIFLLIIVGTASNIGG<br>CSGRPMRQLFNGFENRLPKEPVPPSG<br>PSQCHNALSPYYQTADKFSSTASKVD<br>DYIGCP*
CitIDA2<br>(Ciclev10033211m)<br>(Scaffold 4)	ATGGCTCCGAAAAACATTTTGATCACT<br>TTCCTCTTGTTTCTTTCCCTTCCGCTT<br>CTTCCTGCTCGTTGCATTGCAGCAAAT<br>TATAGAAGCCATAGTTTCTTAAGCCTT<br>CACTACAAAAGCAAGCATAACATGGGT<br>GGCTTTCTGCTGACAAAAGGAGCACCA<br>ATCCCACCTTCAGGACCATCGGGAAGA<br>GGCAACGTCAACCACTCCAAACACGGC<br>CGCTCCGGAAGGCTCAGCCAAGGATGG<br>TGA	MAPKNILITFLLFLSLPLLPARCIAA<br>NYRSHSFLSLHYKSKHNMGGFLLTKG<br>APIPPSGPSGRGNVNHSKHGRSGRLS<br>QGW*
CitIDA3<br>(Ciclev10033191m)<br>(Scaffold 4)	ATGGCTTCTTCTTCTTCTTCTTCTTCT<br>TCTAAGTGTTTGCATATATCATGCAAA<br>CAAATATATCTTCTCTTTCTCATCGTT<br>ATTGTTCTTATTGGTTCTTGCGAGGCG<br>GCTCGGCCCGGCACAACCATGGATTCT<br>GTGAACGTGAAGCTTCACAAGACCAGT<br>TTCAGGTACAAACGGCAGAGGTTCAAC<br>TTCTTGCCTAAAGGGACTCCGATACCG<br>CCTTCTGGTCCATCAAAGAGGCACAAT<br>TCTGTTGTGGATTCTACTCAAAATTGA	MASSSSSSSSKCLHISCKQIYLLFLI<br>VIVLIGSCEAARPGTTMDSVNVKLHK<br>TSFRYKRQRFNFLPKGTPIPPSGPSK<br>RHNSVVDSTQN*
CitIDA4<br>(Ciclev10003011m)<br>(Scaffold 5)	ATGGGTAAAAAGCATCTGATTCTGCTG<br>CTGTGGCTGCTTTTGCTGCTCATGTTT<br>TCGGTTGGGCACACTCACTGCACAAGA<br>TCCTACACTCAAGTTTTCAATATGAAG<br>CAGCCCAGGTCTCATCAGCAGCATTAT<br>TCACCACGTACAATCCTAGGTTTCTTA<br>CCTAAATCATTCCCAATCCCACCTTCT<br>GGTCCTTCCAAGGAACACAACAGCATA<br>GGCTTGCAAAGCTCGCAGAGCTCTCCA<br>TGA	MGKKHLILLLWLLLLMFSVGHTHCT<br>RSYTQVFNMKQPRSHQQHYSPRTILG<br>FLPKSFPIPPSGPSKEHNSIGLQSSQ<br>SSP*
CitIDA5<br>(Ciclev10026873m)<br>(Scaffold 7)	ATGGGTAATTCATGTAGAAGAGTACCT<br>CTAATGATTCTACTTCTATGGTTCATC<br>TTCATCTTCATAACTATTAGCCATTGC<br>CATGGCTCAAGAAGTACTAATGTTTTC<br>AACTTGACGCCAAACTCTCCTCATCAG<br>CAAACAGGCCACTTCTTGGGGTTCTTG<br>CCTAGGCATTTTCCAATACCATCCTCT<br>GGTCCCTCAAGGAAACACAATGACATT<br>GGCTTACAAAATTGGAGATCCCCTTGA	MGNSCRRVPLMILLLWFIFIFITISH<br>CHGSRSTNVFNLTPNSPHQQTGHFLG<br>FLPRHFPIPSSGPSRKHNDIGLQNWR<br>SP*

Supplementary Table 2. Citrus transcripts, CDS and deduced amino acid sequences of the citrus *HSL* genes (source: *Citrus clementina* genomic sequence at Phytozome).

Gene name (Transcript name)	CDS sequence	Peptide sequence
CitHAESA<br>(Ciclev10014127m)<br>(Scaffold 2)	ATGCTTCTGTTTTTCTTCCTGTTATGTCTCACACTCTCCCCGC<br>CCTTGCTCACTCAATCACTGAACCAAGACGGCCTCTACCTCCG<br>GCGAGTCAAACTCGGCCTCTCCGATCCAACTGACTCCCTCGCT<br>TCGTGGGACGACACCCGGGTCGACACCCCGTGTCACTGGCGGG<br>GCGTCTCCTGCGACCCACTCACTCAGCGAGTCACCTCCGTCAA<br>TTTATCCCAGTCGCAGCTCTCCGGCCCTTTCCCCATTTTCTTT<br>TGCCGCCTCCCTTATCTCGCCCAGCTCTCTCTTTACAACAACT<br>ACATCAACTCATCGCTCCCGCTTGACATCTCCACGTGTCGGAA<br>TCTGACTGACTTGAACTTGGGATCCAATTTGCTCGTTGGCGCC<br>ATCCCTGCAACCCTTTCGCAGCTGAAAAACCTCAAGTCCTTAG<br>AACTGCAAGAAAATAACTTCACTGGTGACATCCCTGCGAGTTT<br>TGGTGAGTTCACTCAACTCGAGTCACTCTCACTCTTTAATAAC<br>CTTTTGAATGGAACCATATCCAGTTCGCTCGGCAACATTTCCA<br>CTCTCAAGGAGCTTCGTCTCGCTTACAACCCGTTTCAACCGGG<br>TCAGCTGCCGAGTCAGCTCAGCAACTTGACGAACCTCGAGTAT<br>CTCTGGCTCAGTGGATGTAACCTCCTGGGTGAAATTCCTGAGA<br>GCTTGACTCGGTTGACGAAGCTTAAAAATTTAGACTTGTCGTT<br>CAACGGGCTCACTGGGTCGATTCCGAGTTCGATCACCGAAATG<br>AAAAGCATAGAGCAAATTGAGTTGTTTAAGAACTCGTTGTCTG<br>GCGAATTACCGGTGAAGTGGGTTAATATGACGACGCTTTTGAG<br>GTTTGACGCTTCGATGAATCAGCTGACTGGGACGATTCCGAAC<br>GAGTTGTGTGAGCTACAGCTCGAGTCACTCAACTTGTATGAGA<br>ACAGACTCGAGGGCACTTTGCCAGAGAGTATAGCTCGGTCTAA<br>GAACTTGTCTGAACTCAAATTGTTTAACAACAAACTCAGAGGT<br>GGGCTGCCGAGCGAACTTGGGAAGTACTCTCCTTTAACAACAT<br>TAGACCTCTCGTACAATCAATTTTCTGGTGAAATTCCAGAGGG<br>TTTGTGTGAGAAAGGGTCTTTAGAGGATTTGGTACTGATATAC<br>AATTCATTTTCTGGGAAAATCCCACAAAGTCTAGGAAAATGTC<br>GGAGCTTGAGGAGGGTCCGTTTGAGGCATAACTTGTTATCAGG<br>TTCAGTTCCTGAGATGTTCTGGGGCTTGCCTCATATTTATTTG<br>TTTGAACTTGCTGATAATTCATTTACTGGGAAGATTTCAAAAT<br>CTATTTCTGGTGCAAACAATCTTTCAAGTCTTTTGGTTTCCAG<br>AAATAACTTTTCGGGGTCAATTCCAGATGAAGTTGGGTTGTTG<br>AGTAACTTGGTTGAATTTTCGGGTGATGGTAACAAGTTTGCAG<br>GACAAATTCCAGGGAGTTTAGCGAAATTGAGTCAGTTGGGAAA<br>TCTTGATCTTAGTGAAAATGAACTATCTGGTGGGATTCCAGAA<br>GGAATTGAAAGTTGGAAGAATATCAATGAGCTCAATTTGGCTA<br>ACAATAGGCTCTCTGGTGAAATACCAAGTGAAATTGGGAATTT<br>GCCGGTGCTGAATTATCTTGATCTTTCCGGGAATTTATTTTCT<br>GGGAAAATCCCACTTGAATTGCAGAATTTGAAGCTCAATGTAC<br>TTAATTTGTCAAATAATAGGCTTTCTGGCGAGCTACCTCCTTT<br>ATATGCCAAGGAAATGTATAGGGGTAGCTTCTTGGGGAATCCA<br>GGTTTGTGCGGGGACTTGGCTGGTCTCTGTCCAAAGACTGGTA<br>GATCTAAGAATGAAGGTTCTCTGTGGATTTTTGGATTGATCTT<br>TCTTCTTGCTGGTGTCGTCTTTGTTGTTGGGGTTATTTGGTTC<br>TATGTGAAGTATCGGAAACTTAAGAAGACAAAGAAGGGAATGG<br>CTTTGTCGAAGTGGAAGTCATTCCACAAAATTGGTTTTAGTGA<br>GTTTGAGATTGCTGACTGTCTGAAAGAAGAAAATTTGATTGGA<br>AGTGGAGCTTCGGGGAAAGTTTACAAAGTTTTGCTAAGCAATG<br>GTGATGTAGTGGCAGTGAAGAAACTGTGGGGAAGAGCCAAGAA<br>GGATGATTCAAGTAATGAGTCACAGAGAGATGAATTTGAAGTA<br>GAAGTTGAAACATTGGGAAAGATAAGGCACAAAAATATTGTGA<br>GATTGTGGTGCTGTTGCAACTCCAGAGATTGCAAGCTTCTGGT<br>TTACGAGTACATGCCAAATGGGAGCTTGGGAGATGTGTTGCAT<br>AGCGGCAAAGCAAGTTTGTTGGATTGGCCAACAAGGTATAAGA<br>TAGCTTTGGATGCGGCTGAGGGGTTATCTTATTTGCATCATGA<br>TTGTGTTCCTCCAATTGTTCATCGCGATGTGAAATCCAACAAC<br>ATATTGTTGGATGCAGAGTTTAGTGCTAGAGTTGCAGATTTTG<br>GAGTTGCTAAGGTTGTTGATGGAGTTGGCAAGGGGCCCGAATC<br>CATGTCCGTAATTGCAGGTTCTTGCGGTTACATTGCACCAGAA<br>TATGCATATACTCTTCGAGTGAATGAAAAGAGTGACATTTACA<br>GTTTCGGAGTGGTCCTTCTGGAATTGATTACTGGTAGACCTCC<br>AATTGATCCAGAGTTTGGGGAGAAAGACTTGGTAAAATGGGTC<br>TGCACCACCTTGGACCAGAAAGGCCTGGACAATATAATTGATT<br>CCAACCTCGATTCAAGCTACAAGGATCAAATTTGCAGGGTCCT<br>CGAGATCAGTCTCCTTTGCACCAATGCACTCCCCCTTAATCGC<br>CCCTCCATGCGAAAGGTGGTTAAACTGTTGCAAGAAGCCACCG<br>CAGAGAACAAGTCTAAGACCATCAAGAAAGACGGCAAGCTCTC<br>GCCTTACTACTATGAAGATCCATCTGATAATCAAATTTTAGTT<br>TAA	MLLFFFLLCLTLSPPLLTQSLNQDGLYLRRVKLGLSDPTDSLA<br>SWDDTRVDTPCHWRGVSCDPLTQRVTSVNLSQSQLSGPFPIFF<br>CRLPYLAQLSLYNNYINSSLPLDISTCRNLTDLNLGSNLLVGA<br>IPATLSQLKNLKSLELQENNFTGDIPASFGEFTQLESLSLFNN<br>LLNGTISSSLGNISTLKELRLAYNPFQPGQLPSQLSNLTNLEY<br>LWLSGCNLLGEIPESLTRLTKLKNLDLSFNGLTGSIPSSITEM<br>KSIEQIELFKNSLSGELPVKWVNMTTLLRFDASMNQLTGTIPN<br>ELCELQLESLNLYENRLEGTLPESIARSKNLSELKLFNNKLRG<br>GLPSELGKYSPLTTLDLSYNQFSGEIPEGLCEKGSLEDLVLIY<br>NSFSGKIPQSLGKCRSLRRVRLRHNLLSGSVPEMFWGLPHIYL<br>FELADNSFTGKISKSISGANNLSSLLVSRNNFSGSIPDEVGLL<br>SNLVEFSGDGNKFAGQIPGSLAKLSQLGNLDLSENELSGGIPE<br>GIESWKNINELNLANNRLSGEIPSEIGNLPVLNYLDLSGNLFS<br>GKIPLELQNLKLNVLNLSNNRLSGELPPLYAKEMYRGSFLGNP<br>GLCGDLAGLCPKTGRSKNEGSLWIFGLIFLLAGVVFVVGVIWF<br>YVKYRKLKKTKKGMALSKWKSFHKIGFSEFEIADCLKEENLIG<br>SGASGKVYKVLLSNGDVVAVKKLWGRAKKDDSSNESQRDEFEV<br>EVETLGKIRHKNIVRLWCCCNSRDCKLLVYEYMPNGSLGDVLH<br>SGKASLLDWPTRYKIALDAAEGLSYLHHDCVPPIVHRDVKSNN<br>ILLDAEFSARVADFGVAKVVDGVGKGPESMSVIAGSCGYIAPE<br>YAYTLRVNEKSDIYSFGVVLLELITGRPPIDPEFGEKDLVKWV<br>CTTLDQKGLDNIIDSNLDSSYKDQICRVLEISLLCTNALPLNR<br>PSMRKVVKLLQEATAENKSKTIKKDGKLSPYYYEDPSDNQILV<br>*
CitHAESA-like 1<br>(Ciclev10014138m)<br>(Scaffold 2)	ATGGGACTTCTTACTGGGATGCTAGTATTAGTTGCCTTTCTTT<br>TGTCTCCGCTCCCTTCTCTTTCGCTCAACCAAGAAGGGCTCTA<br>CCTCGAGAGCGTCAAACTGAGCCTCTCAGACCCAGATTCAGCA<br>CTCTCCTCTTGGGGCAGGAACCCGCGGGATGACAGCCCCTGTT<br>CATGGCGCGGTGTCGAGTGCGACCCACGCTCTCACTCCGTCGC<br>CTCTATCGACCTCTCAAACGCCAACATTGCCGGCCCTTTCCCT<br>TCTCTCCTCTGCCGACTTGAAAACCTCACCTTCCTCACTCTCT<br>ACAACAACTCCATCAACTCCACCCTCCCCGACGACATCTCCGC<br>CTGTCAGAATCTCCAACACCTCGACCTCTCCCAGAACTTGCTC<br>ACCGGTACACTCACCCCGGCCTTAGGCGACCTCCCCAACCTGA<br>AGTTTCTCGATTTAACGGGAAACAATTTCTCCGGAGATATTCC<br>GGAGAGTTTCGGCCGGTTTCAGAAGCTTGAAGTCATCTCTCTG<br>GTCTACAATCTTCTCGACGGGACAATTCCCGCATTTCTAGGAA<br>ACATTAGTACGCTCAAGATGTTGAACTTGTCTTACAACCCGTT<br>TCTGCCAGGCCGGATCCCGCCGGAGCTTGGCAATTTGACGAAC<br>CTCGAGATACTCTGGCTCACTGAGTGTAACCTGGTCGGTGAGA<br>TTCCTGACTCCCTCGGCAGACTCGCTAAGCTCGTTGATTTGGA<br>CCTGGCGCTCAACAACTTAGTTGGTGCTATCCCGAGTTCACTC<br>ACTGAGTTGGCCAGTGTCGTCCAGATTGAGCTGTATAACAACT<br>CCCTGACTGGGCACTTGCCGACTGGGTGGTCTAATTTGACGAG<br>CCTGAGACTCCTCGACGCCTCAATGAACGATTTGACTGGGCCG<br>ATTCCTGACGACTTGACTCGGTTGCCACTCGAGAGTCTCAATC<br>TTTACGAAAACCGTTTGGAAGGCAGCTTGCCTGCTACCATTGC<br>TGACTCTCCGGGTTTGTACGAACTCAGGCTTTTTAGAAACCGC<br>CTTAACGGCACCTTACCCGGAGATCTTGGTAAAAACTCGCCGT<br>TGAGATGGGTCGACTTGTCGAACAACCAGTTTACCGGCGAAAT<br>ACCGGCGAGTTTATGCGAAAAGGGGGAGCTGGAGGAGTTGCTG<br>ATGATATACAACTCCTTCACGGGTCAACTACCGGATGGCCTTG<br>GTCATTGCCAGAGCTTGACACGTGTACGGTTGGGGTACAATCG<br>GTTAACCGGTAAGGTGCCACCACTTTTATGGGGTCTCCCTCAC<br>GTATACTTGCTTGAGCTTACTGACAACTTTTTGTCCGGAGAAA<br>TCTCGAAGAATATTGCCGGTGCAGCAAATTTATCGCTTTTGAT<br>TATTTCCAAGAATAACTTAAGTGGATCATTGCCGGAAGAGATT<br>GGTTTCTTGAAAAATCTTGTTGTGCTCTCTGGTAGTGAAAATA<br>AGTTCACTGGGTCACTGCCAGAAAGTTTAACAAATCTTGCTGA<br>GCTGGGAAGTCTTGATCTCCATGCAAATGATTTATCTGGTGAG<br>TTGCCGAGTAGTGTTAGTTCTTGGAAAAAGTTAAACGAGCTTA<br>ATTTAGCAGATAATTTGTTTTATGGGAATATTCCTGAGGATAT<br>TGGGAATTTGTCGGTGTTGAATTACCTGGACTTGTCCAATAAT<br>CGGCTCTCTGGGAGGATCCCTGTTGGGTTACAGAATTTGAAAC<br>TAAATCAGCTGAATGTTTCGAATAATAGGCTTAGTGGGGAGTT	MGLLTGMLVLVAFLLSPLPSLSLNQEGLYLESVKLSLSDPDSA<br>LSSWGRNPRDDSPCSWRGVECDPRSHSVASIDLSNANIAGPFP<br>SLLCRLENLTFLTLYNNSINSTLPDDISACQNLQHLDLSQNLL<br>TGTLTPALGDLPNLKFLDLTGNNFSGDIPESFGRFQKLEVISL<br>VYNLLDGTIPAFLGNISTLKMLNLSYNPFLPGRIPPELGNLTN<br>LEILWLTECNLVGEIPDSLGRLAKLVDLDLALNNLVGAIPSSL<br>TELASVVQIELYNNSLTGHLPTGWSNLTSLRLLDASMNDLTGP<br>IPDDLTRLPLESLNLYENRLEGSLPATIADSPGLYELRLFRNR<br>LNGTLPGDLGKNSPLRWVDLSNNQFTGEIPASLCEKGELEELL<br>MIYNSFTGQLPDGLGHCQSLTRVRLGYNRLTGKVPPLLWGLPH<br>VYLLELTDNFLSGEISKNIAGAANLSLLIISKNNLSGSLPEEI<br>GFLKNLVVLSGSENKFTGSLPESLTNLAELGSLDLHANDLSGE<br>LPSSVSSWKKLNELNLADNLFYGNIPEDIGNLSVLNYLDLSNN<br>RLSGRIPVGLQNLKLNQLNVSNNRLSGELPSLFAKEMYRNSFL<br>GNPGLCGDLEGLCDGRGEEKNRGYVWVLRSIFILAGLVFVFGL<br>VWFYLKYRKFKNGRAIDKSKWTLMSFHKLGFSEYEILDGLDED<br>NVIGSGSSGKVYKVVLSNGEAVAVKKLWRGMSKECESGCDVEK<br>GQVQDQVQDDGFQAEVETLGKIRHKNIVKLWCCCTTRDCKLLV<br>YEYMPNGSLGDLLHSCKGGLLDWPTRYKIIVDAAEGLSYLHHD<br>CVPSIVHRDVKSNNILLDGDFGARVADFGVAKVVDASGKPKSM<br>SVIAGSCGYIAPEYAYTLRVNEKSDIYSFGVVILELVTGRLPV<br>DPEFGEKDLVKWVCSTLDQKGVDHVLDPKLDCCFKEEICKVLN<br>IGLLCTSPLPINRPAMRRVVKLLQEVGTENHSKTGKKDGKLSP<br>YYHEDASDQGSVA*

	ACCGTCTTTGTTTGCGAAAGAAATGTATAGGAATAGCTTTCTT<br>GGTAATCCTGGCTTGTGTGGAGATTTGGAAGGCTTGTGTGATG<br>GTCGTGGTGAAGAGAAAAACAGAGGTTATGTGTGGGTTCTTAG<br>GTCTATTTTTATACTTGCTGGGCTAGTGTTTGTTTTTGGTTTG<br>GTCTGGTTTTACCTGAAGTACAGGAAATTCAAGAATGGAAGAG<br>CCATTGATAAGTCGAAATGGACATTGATGTCTTTCCATAAACT<br>AGGATTTAGTGAGTATGAGATCTTGGATGGTCTCGATGAAGAC<br>AATGTGATTGGAAGTGGATCTTCTGGGAAAGTTTATAAAGTTG<br>TGCTTAGCAATGGAGAGGCTGTTGCTGTGAAGAAGCTATGGAG<br>AGGAATGAGCAAAGAGTGTGAAAGTGGTTGTGATGTTGAGAAA<br>GGTCAGGTCCAAGATCAAGTTCAAGATGATGGGTTTCAAGCTG<br>AGGTTGAGACTTTGGGAAAGATTAGGCACAAGAATATTGTTAA<br>GCTATGGTGTTGCTGTACAACTAGGGATTGCAAGCTTTTGGTC<br>TATGAGTACATGCCAAATGGTAGCCTCGGTGATTTGTTGCATA<br>GTTGTAAAGGAGGCTTGTTAGATTGGCCTACGAGGTACAAGAT<br>AATCGTTGATGCAGCTGAGGGACTTTCGTATTTGCACCATGAT<br>TGTGTTCCTTCAATTGTGCATCGAGATGTTAAATCCAACAATA<br>TATTGTTGGATGGTGATTTTGGTGCACGAGTGGCCGATTTTGG<br>TGTAGCCAAGGTGGTTGATGCCTCTGGAAAGCCTAAATCAATG<br>TCTGTCATTGCTGGTTCCTGTGGTTACATCGCCCCAGAATATG<br>CCTACACACTTCGTGTGAACGAGAAGAGCGACATATACAGTTT<br>CGGAGTAGTCATTCTTGAGTTAGTAACTGGAAGACTGCCAGTC<br>GATCCAGAGTTTGGGGAGAAGGATTTGGTCAAATGGGTCTGCT<br>CTACTTTGGATCAGAAAGGAGTGGACCATGTTCTTGATCCCAA<br>ACTCGATTGCTGTTTTAAAGAAGAAATATGCAAGGTCCTCAAC<br>ATTGGCCTACTCTGCACTAGCCCTCTCCCCATCAACCGCCCTG<br>CAATGAGAAGGGTGGTGAAGTTGTTGCAAGAAGTGGGCACGGA<br>GAACCATTCCAAGACCGGTAAAAAAGATGGGAAGTTGTCACCT<br>TATTACCATGAAGATGCTTCTGATCAGGGAAGTGTAGCTTGA	
CitHAESA-like 2<br>(Ciclev10026946m)<br>(Scaffold 7)	ATGCGATTTCTCGGTGCCAAATCTCTAATAGCACTGTTATTTT<br>CATTCTTGTTATGTTTTTCATTGGCCATATCTCTCCATGGCGA<br>CGCTGAAATCTTGATTCGCGTCAAGAGTGACCAACTTGACGAC<br>CCGAATAGAAAACTTGGCGACTGGGTTCGAACAAGTCAACAGA<br>GTCCATGCAATTGGACTGGCATTACTTGTGAGACTCAGAATCA<br>GTCTGTAGATGGCATTGACCTCTCCGGGTTCGATTTATCGGGT<br>GGGTTTCCTAACGGTTTCTGTCGGATTCGGACACTTCGGAACC<br>TCAATTTATCAGACAATTATTTCAATGGTACTCTCTCTTCTCA<br>GTCTCTATCTCCTTGCTTTCACTTACAAGTCTTAGCACTCGAC<br>TACAACGTATTCATTGGCGAGTTGCCGGATTTCTCCCGAGAAT<br>TTGCTAATTTACAAGTCCTTGATCTTTCTCGCAATAATTTCTC<br>GGGAGATATCCCGGAAAGTTTCGGCCGATTTCCGGTCTTAAAA<br>GTGCTCAACCTTGGTGGAAACTTGCTCAGCGGTTTGATTCCTT<br>CTTTTCTAGGCAATCTGACTGAGTTGACTCACTTCGATCTAGG<br>TTACAATCCCCTGAAGCCCGGTCCATTGCCTTCGAGTGTTGGA<br>AATTTATCAAAGCTTGAAAATTTATGGGCAGCAAAAGCTAATC<br>TCATCGGAGAAATACCAGACTCAATAGGCAAACTCGCTTTTTT<br>ATCAAATCTTGATTTATCAGACAATTTTCTATCCGGGAAAATT<br>CCTCACAGTTTTTCGGGATTGGCAAGTATAGAGCAAATTGAAC<br>TCTTTGACAACCAATTGTCTGGCGAATTACCTGAAAGTTTAAG<br>CAATTTAACTACTCTGCTTAGACTTGATATCTCACAGAACAAT<br>CTCACTGGGAATTTACCAGAGACTATCGCTGCAATGAGTCTCG<br>AATCTTTGAATCTGAACGACAATTACTTTACCGGCGAAATTCC<br>GGAAAGCTTAGCGTCGAATCCCAATCTTGTTCAACTAAAGCTA<br>TTCAACAATAGCTTCAGTGGGAAGTTACCTGATAATCTTGGGA<br>AATATTCGAATTTGGAATATTTCGACGTGTCTACGAATGATTT<br>TACCGGTGCGCTGCCTCGGTTTCTCTGCTTCAGAAACAAGCTT<br>CAGTGCATTATTATATTCAACAACAGGTTCTCAGGGAAAATCC<br>CACAATCGTACGGAGAGTGCAAAACACTGAATTATCTGCGTTT<br>TGGCGGCAACGAACTACAAGGAGAATTACCTAGCAAGTTCTGG<br>GGTCTCCCTGAAGTTGATTTTTTTGAAATGTACAATAATAGAT<br>TTGAAGGTTCGATTTCTCCGTCAATTTCGAACGCTCGCAAGCT<br>CACTGGTATTCTCATCAACGGCAACAACTTTACTGGCGAAGTT<br>CCGTCACAAATATGCACCTTACGTCAACTACAAGCTGTGGATT<br>TAAGCCAAAACCGATTTTCTGGTCACCTGCCGACGTGCATTAC<br>CCAGCTCAACAAGTTACAACAGCTTGAGTTGCAAGAAAACATG<br>TTCACCGGAGAATTGCCACGAAATTTGAACTCACTGACCGCTC<br>TGATTGTGTTGAATCTGTCGACGAACCGGCTTACCGGTACTAT<br>TCCTCCAGAACTTGGAAATTTGGCAGTTTTGACCTCCTTGGAT<br>CTTTCGAGTAACCTGTTGACCGGTGAAATTCCGGTAGAGTTGA<br>CCAAGTTGAAGCTAAACCAGTTCAACATTTCACACAATAAACT<br>CTACGGTGAAGTGCCTTCCGATTTTGATCACGACTTGTTTATT<br>TCTAGTTTATTGGATAACCCGGGTCTCTGCAGCCCGGATTTGA<br>AACCCCTCCCTCCGTGCTCCAAAACCAAACCCGGAACCATTTA<br>CATAGTGGTAATCTTGAGCATTTGCGTCATACTTCTTGTCGGG<br>TGTCTCGTTTGGTTCCTCAAAGTCAAATCCGGATTTTTTAGCA<br>CATCCAAGAGTCCGTGGAAAGTGGTCACATTCCAACGGGTCAG<br>TTTCAATGAAGATGACATACTTCCGCACTTGACAGAACAAAAC<br>CTGATTGGTTCTGGGGGTTCTTGTCGGGTCTATAAAGTGAAGC<br>TGAAGTCTGGTGAAACGGTGGCAGTTAAGAGACTCTTGGGAGG<br>TACTCAGAAACCGGAAACAGAAACCGTCTTCAGGTCCGAAATT<br>GAAACGCTGGGTCGGGTCCGCCATGGCAATGTTGTAAAATTAT<br>TGATGTGCTGTAGCGGTCAAGATTTTAACATTTTGGTGTATGA<br>ATATATGCCAAATGGAAGTTTAGCTGACATGTTGCATGAGAAG<br>GGGCGCTCAGGTTCATTGGATTGGCGCATTCGGTTCTCCATAG<br>CACAAGGTGCAGCTAAAGGGTTGGCATATCTTCATAATGATTG<br>TGTGCCGGCCATCGTGCACCGGGATGTGAAGAGTCACAATATA<br>TTGCTGGACGCCGAGATGGTGCCACGTGTAGCGGATTTTGGGC<br>TTGCGAAGGCGTTGCAGAGTCAAGAGGGGCAGTCTGATGATGC<br>CATGTCGTGTGTTGCTGGGTCCTACGGCTATATCGCGCCTGAG<br>TATGCTTACACAAAGAAAGTGACTGAGAAGAGTGATGTGTACA<br>GCTTTGGCGTAGTGCTGATGGAATTAGTAACTGGTAAAAGGCC<br>GAACGATCCTTCCTTTGGTGAGAATAAGGATATAGTGAGGTGG<br>GTCACGGAGGCAACTTTATCATCTCCTGAGAGAGGTTGCTGCA<br>GAGATTTGAACCAGCTTATTGATCCAAGAATGGATCTATCAAC<br>CTGTGATTATGAAGAAGCTGAGAAGGTTTTGAACGTAGCTCTC<br>ATGTGTACTTCAGATTTTCCCATTAACCGGCCGTCTATGAGAA<br>GAGTAGTCGAATTGCTCCGAGTCGATAAATCATCTCATTGA	MRFLGAKSLIALLFSFLLCFSLAISLHGDAEILIRVKSDQLDD<br>PNRKLGDWVRTSQQSPCNWTGITCETQNQSVDGIDLSGFDLSG<br>GFPNGFCRIRTLRNLNLSDNYFNGTLSSQSLSPCFHLQVLALD<br>YNVFIGELPDFSREFANLQVLDLSRNNFSGDIPESFGRFPVLK<br>VLNLGGNLLSGLIPSFLGNLTELTHFDLGYNPLKPGPLPSSVG<br>NLSKLENLWAAKANLIGEIPDSIGKLAFLSNLDLSDNFLSGKI<br>PHSFSGLASIEQIELFDNQLSGELPESLSNLTTLLRLDISQNN<br>LTGNLPETIAAMSLESLNLNDNYFTGEIPESLASNPNLVQLKL<br>FNNSFSGKLPDNLGKYSNLEYFDVSTNDFTGALPRFLCFRNKL<br>QCIIIFNNRFSGKIPQSYGECKTLNYLRFGGNELQGELPSKFW<br>GLPEVDFFEMYNNRFEGSISPSISNARKLTGILINGNNFTGEV<br>PSQICTLRQLQAVDLSQNRFSGHLPTCITQLNKLQQLELQENM<br>FTGELPRNLNSLTALIVLNLSTNRLTGTIPPELGNLAVLTSLD<br>LSSNLLTGEIPVELTKLKLNQFNISHNKLYGEVPSDFDHDLFI<br>SSLLDNPGLCSPDLKPLPPCSKTKPGTIYIVVILSICVILLVG<br>CLVWFLKVKSGFFSTSKSPWKVVTFQRVSFNEDDILPHLTEQN<br>LIGSGGSCRVYKVKLKSGETVAVKRLLGGTQKPETETVFRSEI<br>ETLGRVRHGNVVKLLMCCSGQDFNILVYEYMPNGSLADMLHEK<br>GRSGSLDWRIRFSIAQGAAKGLAYLHNDCVPAIVHRDVKSHNI<br>LLDAEMVPRVADFGLAKALQSQEGQSDDAMSCVAGSYGYIAPE<br>YAYTKKVTEKSDVYSFGVVLMELVTGKRPNDPSFGENKDIVRW<br>VTEATLSSPERGCCRDLNQLIDPRMDLSTCDYEEAEKVLNVAL<br>MCTSDFPINRPSMRRVVELLRVDKSSH*

Supplementary Table 3. *IDL* gene family of citrus and expressed sequence tags (ESTs) covering the sequence of all citrus *IDL* genes.

Gene name (Transcript name)	EST ID	GenBank ID	Library	Library description
CitIDA1 (Ciclev10017342m)	CS00-C3-700-034-H09-CT.F	EY695297	LIBEST_022237	Peel of fruits, 1 cm in diameter, from *Citrus sinensis* plants grown in the field
	CS00-C3-705-062-D02-CT.F	EY739782	LIBEST_022242	Peel of fruits, 9 cm in diameter, from *Citrus sinensis* plants grown in the field
	CS00-C3-705-088-H01-CT.F	EY741747	LIBEST_022242	Peel of fruits, 9 cm in diameter, from *Citrus sinensis* plants grown in the field
CitIDA2 (Ciclev10033211m)	KN0AAP12YP13	FC928564	LIBEST_022436	Flavedo (the colored portion of fruit peel) and juice vesicles of fruits from *Citrus clementina* plants during phases II and III of development
	C34206F03EF	FC932265	LIBEST_022429	Peel from cold-stored fruits (2ºC) of *Citrus clementina* and from cold-stored fruits infected with the fungus *Penicillium digitatum*
CitIDA3 (Ciclev10033191m)	EST0148	CB250354	LIBEST_012678	Calyx abscission zone (AZ-C) of mature fruits from *Citrus sinensis* plants subtracted with RNA from fruit peel
	KN0AAA1CC04	FC870272	LIBEST_016824	Pedicel abscission zone (AZ-A) and surrounding tissues of flowers from *Citrus clementina* plants grown in the field
CitIDA4 (Ciclev10003011m)	CS00-C3-702-013-A05-CT.F	EY711896	LIBEST_022239	Peel of fruits, 5 cm in diameter, from *Citrus sinensis* plants grown in the field
	CS00-C3-700-046-C02-CT.F	EY696320	LIBEST_022237	Peel of fruits, 1 cm in diameter, from plants of *Citrus sinensis* plants grown in the field
	UCRCS05_0001J05_r	CN185589	LIBEST_015556	Pulp tissue (juice vesicles) from fruits of *Citrus sinensis* plants grown in the field
CitIDA5 (Ciclev10026873m)	CS00-C2-003-020-B06-CT.F	EY685502	LIBEST_022236	Bark (probably with phloem) of shoots from *Citrus sinensis* plants grown in a greenhouse
	CS00-C2-003-006-E06-CT.F	EY686670	LIBEST_022236	Bark (probably with phloem) of shoots from *Citrus sinensis* plants grown in a greenhouse
	CS00-C2-003-042-D05-CT.F	EY686311	LIBEST_022236	Bark (probably with phloem) of shoots from *Citrus sinensis* plants grown in a greenhouse

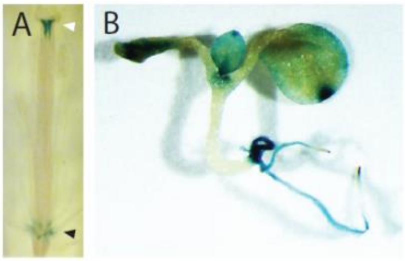

Supplementary Figure 1 *$Pro_{CitIDA3}$:GUS* expression pattern in different tissues of Arabidopsis. (A) Flowers show expression of *CitIDA3* in the style (white arrow head) and floral abscission zone (black arrow head). (B) 1-week-old seedlings; expression in hydathodes, at the base of developing leaves and root.
